# Between Sir Henry Burdett, K.C.B., K.C.V.O., and the Secretary of the National Anti-Vivisection Hospital, Battersea General Hospital (MR. G. W. F. Robbins)

**Published:** 1909-08-28

**Authors:** 


### THE ANTI-VIVISECTION HOSPITAL.

### CORRESPONDENCE

### BETWEEN SIR HENRY BURDETT, K.C.B., K.C.V.O., AND THE SECRETARY OF THE NATIONAL ANTI-VIVISECTION HOSPITAL, BATTERSEA GENERAL HOSPITAL (MR. G. W. F. ROBBINS).

We have received from Mr. Robbins, Secretary of the National Anti-Vivisection Hospital, certain correspondence which has taken place between him and Sir Henry Burdett. On receiving this correspondence we sent a member of the staff to inspect the Anti-Vivisection Hospital in accordance with our practice in these cases, but he was refused admission. We have also obtained copies of the letters omitted by Mr. Robbins from the correspondence.

I.

From Mr. ROBBINS.

National Anti-Vivisection Hospital,
The Battersea General Hospital (The Anti-Vivisection Hospital),
Battersea Park, London, S.W.,
July 29, 1909.

To Sir HENRY BURDETT, K.C.B.

DEAR SIR,—At a meeting of the Council of the Metropolitan Hospital Sunday Fund you are reported (vide *Daily Telegraph* of this date) to have said that the medical profession knew perfectly well that they must employ remedies which would not have been available had it not been for discoveries resulting from vivisection. If you are so correctly reported would you kindly tell me of any remedy which we *must* employ here, and which could *only* have been discovered by vivisectional experiments of animals; and also how you would prove this as a fact?

Yours very faithfully,
(Sgd.) GEORGE W. F. ROBBINS,
Secretary.

---

II.

From Sir HENRY BURDETT.

The Lodge, Porchester Square, W.,
July 30, 1909.

DEAR SIR,—I have received your letter of July 29, which I observe misquotes the report that appeared in the *Daily Telegraph* to which you refer.

Yours faithfully,
(Sgd.) HENRY C. BURDETT.

GEORGE W. F. ROBBINS, Esq.
Secretary, National Anti-Vivisection Hospital,
Battersea General Hospital, Battersea Park, S.W.

III.

From Mr. ROBBINS,

National Anti-Vivisection Hospital and Battersea General Hospital,
Battersea Park, S.W.
July 30, 1909.

To Sir HENRY BURDETT, K.C.B.

DEAR SIR,—I am in receipt of your note, and on comparing my letter with the cutting from the *Daily Telegraph* I find that the words " in the treatment of disease " were omitted. The paragraph was read out to me, and since these words are hardly such as would not necessarily be implied, I feel sure that the chance omission will not cause you to evade the very important and interesting questions I addressed to you.

I think you will find that the following is an absolutely correct quotation of the report, dealing with your remarks. I give the whole paragraph now:

" He hoped its authorities would purge themselves of the pretentious humbug involved in the claims they put forward as to the special advantages of treatment in that hospital from the anti-vivisectionist point of view, because the medical profession knew perfectly well that in the treatment of disease they must employ remedies which would not have been available had it not been for the discoveries resulting from vivisection."

I should be glad, therefore, if you would inform me whether you are correctly reported as above, and, if so, I shall be obliged by your naming any drugs—or one drug—which " must " be used by our " medical " staff, and which " would not have been available had it not been for the discoveries resulting from vivisection." Thirdly, I would ask how you propose logically to prove what you state as fact.

I feel sure that you, in your strong position, will not fail to give me the information for which I ask. Thanking you for pointing out the omission,

I remain, dear Sir,
Yours very faithfully,
(Sgd.) George W. F. Robbins,
Secretary.

IV.

From Sir HENRY BURDETT.
The Lodge, Porchester Square, W.
5th August, 1909.

*Encl.*

Dear Sir,—I am in receipt of your letter of the 30th ultimo, in which it is stated that the paragraph which you incorrectly quoted was read out to you. It is important to get the facts precisely on record, and I therefore venture to ask who dictated your first letter, so that I may know with whom I am dealing in this correspondence?

The omitted words are not trivial, as you indicate, but important. Possibly you may not have read the whole paragraph in the newspapers, except hurriedly. I observe, too, that you invite me to name drugs, though I said nothing about drugs, as such, in my speech, although the evidence before the Royal Commission and elsewhere shows that much might be said on this point. I may further observe that my concern is, and always has been, to secure the maximum efficiency in the administrative, economical, and general management of every voluntary hospital. This is made clear from the fuller summary of my remarks at the Mansion House, published in The Hospital of 31st July, 1909, of which I send you a copy.

Believe me,
Yours faithfully,
(Sgd.) Henry C. Burdett.

Geo. W. F. Robbins, Esq.,
Secretary: National Anti-Vivisection Hospital,
Battersea Park, S.W.

V.

From Mr. ROBBINS.
August 6, 1909.

To Sir Henry Burdett, K.C.B.

Dear Sir,—I am pleased to hear from you again, though we do not seem to be getting on very fast.

You are dealing *with me.*

My first letter was *not dictated* by anybody.

I did not indicate that the words omitted were "trivial," but in my opinion, they were in this case necessarily implied.

However, we have got all now, have we not?

I take it you acknowledge The Hospital's account as a correct report of your words. I asked you to name drugs, or a drug, which must be employed in the treatment of disease in this Hospital (your whole speech was on this Hospital), and which at the same time "would not have been available had it not been for the discoveries resulting from vivisection." I only ask for the *name* of one drug. If you don't want to give it, or cannot, I must do without. If you object to my asking for the name of a drug, "as such," I am quite willing to meet you, and ask you to name a *remedy* which "the medical profession knows," etc., etc. I don't ask for more than its name in the first place. I then asked you (supposing you give me the name of a drug "as such," or remedy "as such"?) to logically prove, by a syllogism if possible, what you state to be a fact—*i.e.*, that the drug (as such) or remedy (as such) would not "have been available had it not been for discoveries resulting from vivisection." I am not now giving any opinion of my own. I am simply asking you to back your assertion by facts and logical argument. If you name a drug (as such) or remedy (as such), and logically prove what I ask, I will tell you frankly whether it "must be" used and is used here. I shall then proceed to ask you, probably, one other very important question, to which I only hope a clear and satisfactory answer to my above questions may bring us speedily. I confess I doubt it being possible.

Believe me, dear Sir,
Very faithfully yours,
(Sgd.) George W. F. Robbins.

*P.S.*—I have no doubt as to your desiring the welfare of Hospitals, etc., etc. But let us keep to the point now at issue—your direct assertions and claim. G. W. F. R.

VI.

From Sir HENRY BURDETT.
The Lodge, Porchester Square, W.
7th August, 1909.

Dear Sir,—I have your letter of the 6th instant, and note that in the postscript you say: "Let us keep to the point now at issue—your direct assertions and claims." As your letter does not fulfil the conditions embodied in your postscript, and as it is my wish to confine this correspondence to the issue I raised, I set it forth here.

I am reported to have said: I hoped the Authorities of the Anti-Vivisection Hospital, Battersea, would purge themselves of the pretentious humbug involved in the claims they put forward as to the special advantages of treatment in that Hospital from the Anti-vivisectionist point of view, because the medical profession knew perfectly well that in the treatment of disease "they" (*i.e.*, the Battersea Hospital) "must employ remedies which would not have been available had it not been for the discoveries resulting from Vivisection." By that statement I stand, for it is demonstrably true. If this correspondence is to proceed further I must ask you to reply to the following questions:—

1. Is the aseptic system adopted by the medical staff of the Anti-Vivisection Hospital, and are antiseptics used in surgical cases treated there?

2. Are cases of enteric fever and diphtheria admitted for treatment to your Hospital, and do you admit diseases caused by specific micro-organisms?

I may point out in this connection that within the last eighteen months Dr. Wall, when asked: "Do you (the Anti-Vivisection Hospital) test the sputum for tubercle bacilli in cases of doubtful phthisis?" replied, "Most certainly we do." My statement at the Mansion House expressed the hope that the Authorities of your Institution would "mend their ways, purge their methods, and, in fact, fall into line with all the best administered hospitals in the country." If, as I desire to believe, that is their object, you will tersely reply to the questions I have asked, and so confine this correspondence, as it must be confined as far as I am concerned, to the single issue I raised, as set forth above.

I am, yours faithfully,
(Sgd.) Henry C. Burdett.

George F. Robbins, Esq.

VII.

From Mr. ROBBINS.
August 9, 1909.

To Sir Henry Burdett, K.C.B.

Dear Sir,—From your letter I gather that you now desire to name (*a*) the aseptic system, (*b*) the use of antiseptics in surgical cases, as remedies (as such) which the Battersea Hospital "must employ," and "*which would not have been available had it not been for the discoveries resulting from Vivisection.*"

It now rests with you, as directly challenged by me to *prove* this assertion (in inverted commas and underlined above) to be a fact, in the case of each of these remedies (*a*) and (*b*).

That is your own point, chosen from your speech by me, and on which I challenged you, and I await your proof, which is all I ask or care for at present.

Yours very faithfully,
(Sgd.) George W. F. Robbins,
Secretary.

*P.S.*—Certainly let us keep to this single issue. I have no intention of wandering into *opinions*, etc. I want the fact, as you claim it, *proved.* G. W. F. R.

VIII.

From Sir HENRY BURDETT.
The Lodge, Porchester Square, W.,
August 11, 1909.

George W. F. Robbins, Esq.

Dear Sir,—May I point out that you have not answered the two simple questions I asked you in my last letter. If you are, as your postscript declares, willing to keep to this single issue and want the facts to be proved, you will not delay to send me plain answers to my questions. In any case please understand that, if this correspondence is to proceed further, you must now answer directly questions 1 and 2 of my letter of the 7th inst.

I am, yours faithfully,
(Sgd.) Henry C. Burdett.

IX.

From Mr. ROBBINS.
12th August, 1909.

To Sir Henry Burdett, K.C.B.

Dear Sir,—I must decline to help a gentleman in your position to prove a statement made by you, freely reported in many newspapers, and correctly at least (as you assure me, I understand?) in The Hospital—a copy of which you sent me. To prove your assertion is, I maintain, an impossibility. If your assertion that it is "demonstrably true" is correct, the "onus probandi" rests with *you.*

The result appears now to be, as I fully anticipated—*nil.* When you have proved that the two remedies named by yourself, *i.e.* (*a*) the Aseptic System; (*b*) the use of Antiseptics in surgical cases—"would not have been available, had it not been for the discoveries resulting from vivisection," we may proceed.

This is a perfectly fair demand, or challenge, and I who make it shall adhere to it. Until you prove your claim I am content to record this as another failure to justify the claims of Vivisectionists.

This letter closes my part of this correspondence, until I receive your attempt at proof, which I hope will be terse.

This would not be the first time that a gentleman has, on a Metropolitan Hospital Sunday Fund platform, made assertions which he could not prove, and for making which he even declined to give his authority.

Believe me, dear Sir,
Yours very faithfully,
(Sgd.) George W. F. Robbins,
Secretary.

I propose to send this correspondence to the press.—G. R.

X.

From Sir HENRY BURDETT.
The Lodge, Porchester Square, W.,
August 15, 1909.

Dear Sir,—The result of our correspondence so far is by no means "nil," as you state.

The position you have taken up in refusing to answer two simple questions which the authorities of every reputable Voluntary Hospital throughout the country would have answered without hesitation in course of post leaves your Institution in an invidious position.

In the absence of official information until an authoritative answer is forthcoming to my questions no prudent person of either sex can surely attend your institution as a patient or permit any member of their family to do so.

I need no help of yours to prove my statement, but certain changes at your institution compel me to ask you to state precisely where your Hospital stands to-day in regard to the material matters of treatment covered by my questions.

Yours faithfully,
(Sgd.) Henry C. Burdett.

George W. F. Robbins, Esq.

XI.

From Mr. ROBBINS.

National Anti-Vivisection Hospital and Battersea General Hospital, Battersea Park, S.W.,

August 16, 1909.

My Dear Sir,—I am more than surprised at your letter.

I now gather that you were, knowingly, straying from the points at issue (*i.e.*, my challenge of your statement and your ability to prove it), and were asking your " simple questions "—simple enough, I quite allow in themselves—for quite extraneous objects and purposes of your own; for you admit that they were quite unnecessary for your proof. Then, Sir, why ask them in this connection?

I now invite you for the last time—nay, as far as I can, I *demand* it—to prove your statement, and to drop all this seeming evasion of a perfectly straightforward and legitimate challenge, which you undoubtedly accepted, and declare you can meet without the assistance which you have asked.

What I declined was, to help you in your, as I regard it, dilemma.

After your last letter and your discourteous treatment of this Hospital, no further letter from you will receive attention, unless it contains your attempt at proof—I have not time to give you for any other purpose.

Believe me, dear Sir,

Very faithfully yours,

(Sgd.) George W. F. Robbins,
Secretary.

To Sir Henry Burdett, K.C.B.

